# How COVID-19 induces cytokine storm with high mortality

**DOI:** 10.1186/s41232-020-00146-3

**Published:** 2020-10-01

**Authors:** Shintaro Hojyo, Mona Uchida, Kumiko Tanaka, Rie Hasebe, Yuki Tanaka, Masaaki Murakami, Toshio Hirano

**Affiliations:** 1 Molecular Psychoimmunology, Institute for Genetic Medicine, Graduate School of Medicine, Hokkaido University, Hokkaido, 060-0815 Japan; 2 Headquarters, National Institutes for Quantum and Radiological Science and Technology, Chiba, 263-8555 Japan

## Abstract

The newly emerging coronavirus disease 2019 (COVID-19) caused by severe acute respiratory syndrome coronavirus 2 (SARS-CoV-2) was first reported in Wuhan, China, but has rapidly spread all over the world. Some COVID-19 patients encounter a severe symptom of acute respiratory distress syndrome (ARDS) with high mortality. This high severity is dependent on a cytokine storm, most likely induced by the interleukin-6 (IL-6) amplifier, which is hyper-activation machinery that regulates the nuclear factor kappa B (NF-κB) pathway and stimulated by the simultaneous activation of IL-6-signal transducer and activator of transcription 3 (STAT3) and NF-κB signaling in non-immune cells including alveolar epithelial cells and endothelial cells. We hypothesize that IL-6-STAT3 signaling is a promising therapeutic target for the cytokine storm in COVID-19, because IL-6 is a major STAT3 stimulator, particularly during inflammation. We herein review the pathogenic mechanism and potential therapeutic targets of ARDS in COVID-19 patients.

## Background

Coronavirus disease 2019 (COVID-19), which is caused by severe acute respiratory syndrome coronavirus 2 (SARS-CoV-2), has globally spread to an ongoing pandemic since the first case of infection was reported in 2019. Patients with poor prognostic features upon hospital admission frequently encounter complications with significant mortality, particularly by acute respiratory distress syndrome (ARDS) with a broad spectrum of diseases such as multiorgan failure, and blood clots [1]. No effective vaccine strategy or approved medication for the treatment of this contagious disease has been established, although clinical trials are intensively being performed (https://clinicaltrials.gov/ct2/who_table).

Accumulating evidence suggests that the severity of COVID-19 is associated with an increased level of inflammatory mediators including cytokines and chemokines such as interleukin (IL)-2, IL-7, IL-10, tumor necrosis factor (TNF), granulocyte colony-stimulating factor (G-CSF), monocyte chemoattractant protein-1 (MCP1; also known as CCL2), macrophage inflammatory protein 1 alpha (MIP1α; also known as CCL3), CXC-chemokine ligand 10 (CXCL10), C-reactive protein, ferritin, and D-dimers in blood upon SARS-CoV-2 infection [2–10]. Of note, among the elevated inflammatory mediators, the blood IL-6 level is highly correlated with the disease mortality when COVID-19 survivors and non-survivors are compared [1, 11], suggesting that fatal COVID-19 is characterized as a cytokine release syndrome (CRS) that is induced by a cytokine storm with high mortality [12–14]. Thus, IL-6 serves as a possible mechanism of treatment for severe COVID-19 patients, raising the possibility that one therapeutic option for the disease may be targeting excessive inflammation caused by IL-6 receptor (IL-6R) signaling with monoclonal antibody therapy or treatment with chemical modulators to block the signaling cascade while maintaining a sufficient antiviral primary immune response. In this regard, the use of two clinically approved IL-6R antagonists, tocilizumab (TCZ) and sarilumab (SAR), which are currently used for the treatment of rheumatoid arthritis, could be expected to play a crucial part in the treatment for severely ill patients. With this in mind, here we discuss the potential pathogenetic mechanisms and therapeutic options for COVID-19, focusing on IL-6-signal transducer and activator of transcription 3 (STAT3) signaling.

## Viral entry of SARS-CoV-2

**Fig. 1:**
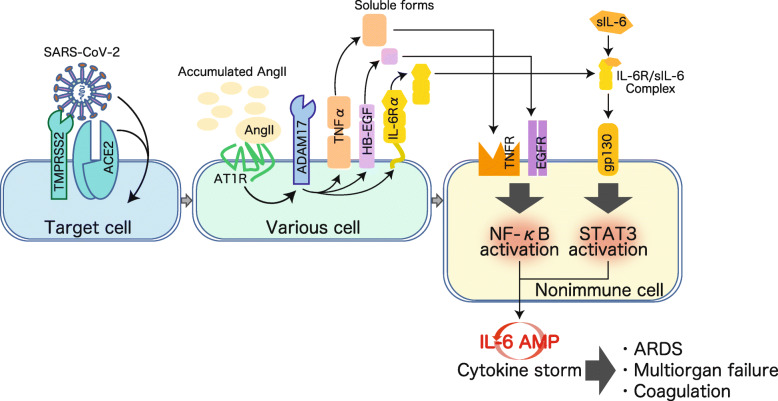
IL-6-STAT3 signaling is a potential therapeutic target for COVID-19 mediated by cytokine storm. SARS-CoV-2 enters cells via cell membrane-localized ACE2 depending on TMPRSS2 and CatB/L activities for viral S protein priming. The viral pathogen-associated molecular patterns trigger IL-6 production followed by activation of the NF-κB pathway via PRRs in both immune and non-immune cells, leading to an inflammatory response. On the other hand, upon the occupancy of ACE2 by SARS-CoV-2, the increased serum level of free Ang II due to a reduction of ACE2-mediated degradation also promotes activation of the NF-κB pathway via AT1R, followed by IL-6 production. Simultaneously, Ang II-AT1R signaling activates ADAM17 and ADAM10 protease activity, and the resulting production of TNF-α, sIL-6Rα, and EGF initiates the TNFR-NF-κB, IL-6R-STAT3, and EGF-NF-κB signaling pathways. Consequently, the concomitant inflammatory cascades of NF-κB- and STAT3-mediated signaling further augment NF-κB activity and establish an inflammatory circuit, the IL-6 amplifier (IL-6 AMP), which describes an IL-6-based positive feedback loop for inflammation in non-immune cells. Thus, the cytokine storm caused by the hyper-activation of NF-κB in IL-6 AMP may cause fatal symptoms such as ARDS, severe pneumonia, multiorgan failure, and coagulation in a subgroup of hospitalized COVID-19 patients. In line with this, the blockade of IL-6-STAT3 signaling should shed light on the treatment of severe COVID-19

Within the past two decades, severe respiratory diseases were caused by zoonotic infections of SARS-CoV and MERS-CoV from animals to humans in endemic areas. In late December 2019 in Wuhan City, China, SARS-CoV-2, belonging to the novel RNA *Betacoronavirus*, was discovered as a causative agent of COVID-19, which has currently become a worldwide pandemic. Genomic analysis revealed that SARS-CoV-2 shares about 80% genetic identity with SARS-CoV, around 50% with MERS-CoV, and around 90% with bat-SL-CoVZC45 and bat-SL-CoVZXC21 coronaviruses, suggesting bat-to-human zoonotic transmission of this new virus [15, 16]. Furthermore, proteomic characterization also showed that SARS-CoV-2 has seven conserved non-structural domains similar to SARS-CoV, suggesting a relationship between the two *Betacoronaviruses*. Despite amino acid differences with SARS-CoV, SARS-CoV-2 has a similar receptor-binding domain to that of SARS-CoV by utilizing angiotensin-converting enzyme II (ACE2) for host cell entry (Fig. 1) [17, 18]. In fact, two independent research groups have provided evidence that SARS-CoV-2 requires ACE2 to infect host cells [16, 19)]. Zhou et al. revealed that SARS-CoV-2 can enter cells expressing ACE2 originated from humans, Chinese horseshoe bats, civet, and pigs [16]. Also, it cannot enter cells expressing either dipeptidyl peptidase 4 or aminopeptidase N, the entry receptors for MERS-CoV and HCoV-229E, respectively. SARS-CoV-2 entry via human ACE2 is dependent on transmembrane serine protease 2 (TMPRSS2) and the endosomal cysteine proteases cathepsin B and L (CatB/L) for viral spike (S) protein priming [19]. TMPRSS2 is necessary for cleaving the viral envelope-located trimeric S protein at the S1/S2 and the S2’ sites, leading to the fusion of the viral and cellular membranes mediated by the S2 subunit of S protein after the engagement of the S1 subunit to the cell surface receptor and for the subsequent viral internalization in the pulmonary epithelium [19]. Notably, ammonium chloride, an inhibitor of CatB/L, inhibited SARS-CoV-2-S protein-driven entry into 293 T cells (TMPRSS2-negative) expressing ACE2, but less so into Caco-2 cells (TMPRSS2-positive). A clinically proven TMPRSS2 inhibitor, camostat mesylate (NCT04321096), which is approved for clinical use in Japan to treat pancreatic inflammation, partially prevented SARS-CoV-2-driven entry into Caco-2 cells, but together with E-64d, an inhibitor of CatB/L completely inhibited the entry. In addition, the forced expression of TMPRSS2 rescued the SARS-CoV-2-S-dependent entry into CatB/L-suppressed 293 T cells, suggesting that the entry of SARS-CoV-2 is induced when cells express TMPRSS2 regardless of CatB/L expression and that SARS-CoV-2 cell entry depends on surface ACE2 and TMPRSS2 molecules. Thus, ACE2 and TMPRSS2 could be predominant targets for COVID-19 therapeutics. Of note, despite the genetic identity of the receptor-binding domain (RBD) structure between SARS-CoV and SARS-CoV-2, several reports have shown anti-RBD monoclonal antibodies cannot neutralize SARS-CoV-2 [20, 21], suggesting that cross-neutralization protection between the two viruses is limited. Moreover, SARS-CoV-2 can infect cells that have Fc receptors (FcRs), which provide the ability of antibody-mediated internalization in macrophages, monocytes, or B cells even without ACE2 and TMPRSS2 expression particularly during the later time point after infection [22].

## Hyper inflammatory pathogenesis of COVID-19

Among coronaviruses in humans, HCoV-229E, HCoV-NL63, HCoV-OC43, and HCoV-HKU1 cause mild cold symptoms upon infection in the upper respiratory tract [23]. On the other hand, SARS-CoV, SARS-CoV-2, and MERS-CoV cause fatal pneumonia in some cases after replicating in the lower respiratory tract. Following the infection of SARS-CoV-2 in patients, symptoms occur within 5–6 days when the viral load reaches a peak, and 97.5% of symptomatic patients further develop COVID-19 within 2 weeks [2, 7, 9, 24–26]. Similar to SARS- and MERS-CoV-induced symptoms, a subgroup of patients admitted to hospitals exhibit severe respiratory failure with dyspnea as observed in chest CT scans, bilateral lung infiltration, lymphopenia, muscle/joint pain, headache/dizziness, diarrhea, nausea, and coughing up of blood [3, 4, 6, 8, 10, 27–29]. Notably, severe COVID-19 is associated with mortality and poor prognostic features even when patients are hospitalized [30]. The wide spectrum of the disease severity for these hospitalized patients includes not only pneumonia, severe damage to the airways, and pulmonary edema, but also ARDS, which is a causative syndrome of death in 70% of fatal COVID-19 cases, in which aggressive inflammatory responses occur [6, 31].

Upon viral infection, alveolar epithelial cells, macrophages, and blood circulating monocytes are activated via toll-like receptors as pattern recognition receptors (PRRs) by the virus products and produce a robust amount of inflammatory cytokines and chemokines, which attract more immune cells, in particular, monocytes and T cells, resulting in widespread lung inflammation (Fig. 1). The postmortem pathology of COVID-19 patients shows interstitial mononuclear inflammatory infiltrates dominated by lymphocytes in the lung and severe lymphopenia with hyperactivated T cells in the peripheral blood [32]. Furthermore, COVID-19 patients also have lower levels of regulatory T cells, which are more obviously decreased in severe cases [33]. Hospitalized patients with severe COVID-19 show high levels of IL-2, IL-7, IL-10, G-CSF, TNF, CXCL10, MCP1, and MIP1α in serum [6], suggesting that severe COVID-19 is dictated as a cytokine release syndrome (CRS), which is a disorder induced by cytokine storms [12–14]. Among the elevated levels of inflammatory mediators in COVID-19 patients, the blood levels of IL-6 are noticeably higher in non-survivors compared to survivors [1, 2, 11, 34] and predict the need for mechanical ventilation [35].

These findings have led to the hypothesis that the main cause of death of COVID-19 is ARDS with cytokine storms. Notably, intravascular coagulation is one of the causes of multiorgan injury, which is mainly mediated by inflammatory cytokines, in particular, IL-6 [36–38]. Patients exhibit multiorgan failure with coagulation abnormalities represented by lower platelets count and increased D-dimer, which are increasingly associated with poor prognosis and explain the microthrombi of the lungs, lower limbs, hands, brain, heart, liver, and kidneys [39–42]. Similar observations are seen in most patients with SARS-CoV infection who progressed to renal failure [2, 43]. Another reason of multiorgan failure is that SARS-CoV-2 infection in endothelial cells also causes cell death, which leads to vascular leakage and induces a cytopathic effect on airway epithelial cells [44].

Thus, it would seem that the disease severity or mortality comes from cytokine storms including ARDS triggered by viral lung infection, which accounts for multiorgan failure across the body [45]. These inflammatory mediators can also lead to vascular hyperpermeability and stimulate endothelial cells that express ACE2 on arteries and veins that together with viral particles cause systemic inflammation [46, 47].

## IL-6-STAT3 signaling as a potential cause of the ARDS via cytokine storms in COVID-19 patients

### IL-6 amplifier, machinery for excessive inflammation

SARS-CoV-2 infection induces the endocytosis of ACE2 together with SARS-CoV in target cells including epithelial cells and endothelial cells, resulting in an increase of serum angiotensin II (Ang II) levels due to the reduction of ACE2 surface expression (Fig. 1) [17, 48]. Ang II increment is also obtained in lung-injury models, in which ACE2 is dramatically reduced upon acid treatment [49]. Ang II acts not only as a vasoconstrictor but also as a pro-inflammatory cytokine via Ang II type 1 receptor (AT1R) [50]. Therefore, it is hypothesized that a renin-angiotensin system (RAS) may be involved in the ARDS development following SARS-CoV-2 infection [12]. In fact, treating mice with AT1R inhibitors or exogenous recombinant ACE2 suppresses ARDS development induced by SARS-CoV infection [17]. In addition, a possible benefit of RAS inhibitors in COVID-19 patients has been reported [51–53]. The Ang II-AT1R signaling axis activates ADAM metallopeptidase domain 17 (ADAM17), which in turn digests the membrane forms of epidermal growth factor family members (EGF, epiregulin, amphiregulin, transforming growth factor-alpha, etc.) and TNF-α, all of which stimulate the NF-κB pathway (Fig. 1) [12, 50, 54]. ADAM17 is also an enzyme that processes membrane-bound IL-6Rα to the soluble form (sIL-6Rα) together with ADAM10. Therefore, we hypothesize serum Ang II and sIL-6Rα might be predictive markers of COVID-19 severity.

Once sIL-6Rα is generated, the sIL-6R-IL-6 complex transduces intracellular signaling via its binding to gp130, a signal transducer of IL-6, which is expressed on non-immune cells including endothelial cells, epithelial cells, and fibroblasts even without membrane IL-6R expression, followed by the activation of Janus kinase (*JAK*)/*STAT3* [55]. Thus, Ang II-AT1R signaling can create an IL-6-mediated positive feedback loop of NF-κB signaling, a mechanism known as the IL-6 amplifier, during lung inflammation followed by ARDS with multiorgan failure and coagulation (Fig. 1).

The IL-6 amplifier is a hyper NF-κB activation machinery in non-immune cells induced by the simultaneous activation of NF-κB and STAT3. It induces a massive and sustained production of NF-κB target genes, including IL-6, chemokines, and growth factors, which is critical for the development of various disease models including lung transplantation, rheumatoid arthritis, and multiple sclerosis [56–60]. Furthermore, we have shown that the co-activation of NF-κB and STAT3, which is evidence of activation of the amplifier, is observed in clinical specimens from patients with inflammatory diseases [56, 61]. Additionally, the expression of target molecules of the inflammation amplifier is higher in the serum of patients with rheumatoid arthritis or multiple sclerosis [56, 61]. Moreover, the amplifier activation depends on the concentrations of NF-κB stimulators and of IL-6 around non-immune cells, but these concentrations vary between cells. Indeed, activation tends to occur more easily in tissue-specific non-immune cells such as tracheal basement cells, synovial fibroblasts, keratinocytes, kidney tubule cells, and chondrocytes. Therefore, through the IL-6 amplifier, these cells could regulate several tissue specific-inflammatory diseases [56–60]. Furthermore, activation of the IL-6 amplifier depends on various environmental and genetic factors. Moreover, we have reported that stress and pain can be triggered for the activation of the IL-6 amplifier at specific blood vessels [62, 63], and some SNPs have an effect on the activation, particularly via the NF-κB pathway [56, 61]. These factors could explain the mild to severe COVID-19 phenotypes observed.

### Possible therapeutics for COVID-19

As mentioned above, excessive IL-6 levels are highly correlated with the lethal complications of COVID-19 patients [16, 64, 65]. Notably, a previous report showed that inhibition of the NF-κB pathway in animals infected with SARS-CoV decreases mortality and IL-6 levels [66].

As described above, the IL-6 amplifier plays a critical role in chronic inflammatory diseases. The activation of the IL-6 amplifier may induce a cytokine storm, a phenotype of dysregulated inflammation. If this is the case, the cytokine storm in severe COVID-19 could be inhibited by blockade of the IL-6 amplifier [12]. Consistently, chimeric antigen receptor (CAR)-T cell-induced fatal cytokine storm was prevented by an IL-6-STAT3 blocker [11, 12, 67].

Because the IL-6 amplifier is activated by the coactivation of NF-κB and STAT3 in non-immune cells, NF-κB and STAT3 should be potential regulators of the COVID-19-mediated cytokine storm shown in Fig. 1. There are many NF-κB activators, such as PRRs, AT1R, ADAM17/10, TNF-α-TNFR, and EGF-EGFR. Some could be therapeutic targets for the cytokine storm. However, IL-6 is the major STAT3 activator during inflammatory responses. Considering the availability of IL-6 inhibitors, IL-6-STAT3 blockade could be a simple option to prevent COVID-19-induced cytokine storm. TCZ is a recombinant humanized monoclonal anti-IL-6R antibody and currently approved for use in patients with CAR-T cell-induced severe cytokine storm or CRS with fever, hypoxia, acute renal failure, hypotension, and cardiac arrhythmia that often warrants ICU admission [11, 67]. The effectiveness of TCZ has been also reported in CRS related to several other conditions, such as sepsis, graft-versus-host disease, and macrophage activation syndrome [68–70]. Common side effects of TCZ include increased upper respiratory tract infections. Therefore, it might be difficult to use TCZ for COVID-19 patients at the early phase of the disease. Nevertheless, the inhibition of the signal transduction mediated by IL-6, the critical STAT3 inducer for the IL-6 amplifier, through binding to both mIL-6Rα and sIL-6Rα is a reasonable approach to consider for treating cytokine storm in COVID-19 patients [12, 55]. In fact, Luo and colleagues reported that TCZ administration stabilizes clinical outcomes with a reduction of increased C-reactive protein levels in more than half of COVID-19 patients at risk of a cytokine storm [71]. Moreover, Xu et al. investigated whether TCZ treatment successfully improves the respiratory function in a total of 21-hospitalized COVID-19 patients. Indeed, among these 21 patients, 20 patients were recovered after the TCZ therapy [72]. Toniati et al. also showed that 77% of patients out of 100 rapidly improved clinical and respiratory status after TCZ treatment [73]. These series of study suggest that IL-6-STAT3 signaling could be a promising target for the clinical intervention of COVID-19.

Several ongoing clinical trials for TCZ in COVID-19 patients have been documented (phase II; NCT04317092, NCT04445272, NCT04377659, phase III; NCT04320615, NCT04330638, NCT04345445) (https://clinicaltrials.gov/ct2/results?cond=COVID-19). Another anti-IL-6R antibody, SAR, used for rheumatoid arthritis [74], has been tested in a multicenter, double-blind, clinical phase II/III study in patients with severe COVID-19 (NCT04315298) [75]. Although Roche has not reached significant effects of TCZ in phase III study (NCT04320615), more careful study for patient selection is important. We hypothesize that the timing of the treatment should be important for suppressing the cytokine storm induced by SARS-CoV-2 infection. Because patients with severe COVID-19 symptoms have multiorgan failure due to the high expression level of cytokines, some of which regulate the function of the organs directly or indirectly via blood endothelial cells that express ACE2, we hypothesize that IL-6 inhibitors including TCZ could mitigate cytokine storms in COVID-19 patients before multiorgan failure. As described in the review, the study that found IL-6 inhibitors could suppress cytokine storms in patients after CAR-T cell therapy most likely did so by blocking the IL-6 amplifier [67]. Indeed, on 18th September 2020, phase III study met its primary endpoint, showing that patients with COVID-19 associated pneumonia who received TCZ were 44% less likely to progress to mechanical ventilation or death compared to patients who received placebo. Other therapeutic options that potentially inhibit JAK kinases related to IL-6 signaling, such as baricitinib (phase II/III; NCT04340232) [76], are also potential therapeutic candidates.

## Conclusions

The cytokine storm and CRS in fatal COVID-19 are represented by several pathological features such as ARDS, coagulation, and multiorgan dysfunctions. Since blood IL-6 levels are highly correlated with the lethal complications of COVID-19, we propose that IL-6 plays a pivotal role in the disease augmentation and can be thus a useful biomarker for determining the disease severity. With this regard, TCZ is a good therapeutic option for disrupting the IL-6 amplifier, an IL-6-mediated hyper-inflammatory mechanism that can be triggered by innate immune signaling upon viral infection together with Ang II-AT1R-mediated signaling due to the reduction of Ang II-quencher ACE2. However, in general, monoclonal antibody-based therapeutics impose a substantial cost burden on patients and society. Therefore, future research should be directed to the identification of responsible molecules that regulate the IL-6 amplifier in severe COVID-19, thereby enabling the development of new clinical interventions using small molecules against these targets. Intriguingly, SARS-CoV, MERS-CoV, and SARS-CoV-2 show high genomic diversity and are considered to have originated from bats. Given that these viruses share viral spike proteins and utilize human ACE2 receptor for their entry, it is highly possible that a novel bat coronavirus may have been transmitted to humans to cause emerging infectious diseases. Therefore, clarification of the molecular mechanism for how CRS promotes multiple symptoms in coronavirus-related diseases is needed. Better understanding of this issue will facilitate the development of novel therapies in preparation for future COVID outbreaks.

## Data Availability

Not applicable.

## Abbreviations

ACE2: Angiotensin-converting enzyme II
ADAM17/10: ADAM metallopeptidase domain 17/10
Ang II: Angiotensin II
ARDS: Acute respiratory distress syndrome
AT1R: Ang II type 1 receptor
CAR: Chimeric antigen receptor
Cat: Cathepsin
COVID-19: Coronavirus disease 2019
CRS: Cytokine release syndrome
CXCL10: CXC-chemokine ligand 10
EGF: Epidermal growth factor
G-CSF: Granulocyte colony-stimulating factor
HCoV: Human coronavirus
ICU: Intensive care unit
IL: Interleukin
MCP1: Monocyte chemoattractant protein-1
MIP1α: Macrophage inflammatory proteins 1-alpha
MERS-CoV: Middle East respiratory syndrome
PRR: Pattern recognition receptor
RBD: Receptor-binding domain
SAR: Sarilumab
SARS-CoV: Severe acute respiratory syndrome coronavirus
STAT3: Signal transducer and activator of transcription 3
TCZ: Tocilizumab
TMPRSS2: Transmembrane serine protease 2
TNF: Tumor necrosis factor
